# Integrating open- and closed-ended questions on attitudes towards outgroups with different methods of text analysis

**DOI:** 10.3758/s13428-023-02218-x

**Published:** 2023-10-16

**Authors:** Karolina Hansen, Aleksandra Świderska

**Affiliations:** Faculty of Psychology, University of Warsaw, Stawki 5/7, 00-183 Warsaw, Poland

**Keywords:** Open-ended, Closed-ended, Natural language, Text analysis, Meaning extraction, Sentiment analysis, Attitudes

## Abstract

Researchers in behavioral sciences often use closed-ended questions, forcing participants to express even complex impressions or attitudes through a set of predetermined answers. Even if this has many advantages, people’s opinions can be much richer. We argue for assessing them using different methods, including open-ended questions. Manual coding of open-ended answers requires much effort, but automated tools help to analyze them more easily. In order to investigate how attitudes towards outgroups can be assessed and analyzed with different methods, we carried out two representative surveys in Poland. We asked closed- and open-ended questions about what Poland should do regarding the influx of refugees. While the attitudes measured with closed-ended questions were rather negative, those that emerged from open-ended answers were not only richer, but also more positive. Many themes that emerged in the manual coding were also identified in automated text analyses with Meaning Extraction Helper (MEH). Using Linguistic Inquiry and Word Count (LIWC) and Sentiment Analyzer from the Common Language Resources and Technology Infrastructure (CLARIN), we compared the difference between the studies in the emotional tone of the answers. Our research confirms the high usefulness of open-ended questions in surveys and shows how methods of textual data analysis help in understanding people’s attitudes towards outgroup members. Based on our methods comparison, researchers can choose a method or combine methods in a way that best fits their needs.

Should the UK leave EU? Should Poland let the refugees in? At first glance, a yes/no question in a poll would suffice to assess people’s opinions or predict the results of a referendum. However, asking about conditions under which the UK should leave the EU or Poland should accept refugees could allow for a better understanding of people’s attitudes. In the current research, we provide examples and guidance on what methods to use in the study of attitudes towards outgroups, focusing in particular on refugees as the example of an outgroup. We are comparing and integrating different methods as well as different approaches and tools for analysis of the written responses provided by participants: manual content analysis and three tools for automated text analysis (Meaning Extraction Helper, MEH; Linguistic Inquiry and Word Count, LIWC; and Sentiment Analyzer from the Common Language Resources and Technology Infrastructure, CLARIN).

## Measuring attitudes towards outgroups

Most of the studies in behavioral sciences in general, and more specifically those measuring attitudes (to refugees, immigrants, climate change, and many others), use a top-down approach (e.g., Bansak et al., 2016; Esses et al., 2013; Wike et al., 2016). Within a top-down approach, researchers rely on existing theories that describe the relationships between specific variables to determine how these variables should be assessed (e.g., Forman et al., 2008). Such assessment is typically based on closed-ended questions, whereby researchers present participants with statements about a matter of interest and participants select an answer from predetermined options (Forman et al., 2008; Baburajan et al., 2020). In psychology, responses on a rating scale are especially common (Krosnick, 1999; Preston & Colman, 2000).

Relatively few studies let participants express what they think in their own words. This is possible by asking open-ended questions, which is characteristic of qualitative research (Forman et al., 2008). Responses to open-ended questions are then analyzed inductively within a bottom-up approach, that is, the researchers start with what is in the data to get to more abstract findings (Forman et al., 2008). The main benefit of using open-ended questions in research is that participants’ responses are freely constructed rather than suggested by the options provided by the researcher. This generates data that otherwise might not be possible to obtain from theory and the researchers' reasoning (e.g., Haddock & Zanna, 1998). Furthermore, previous research shows that by using open-ended questions researchers can better understand people's opinions (Geer, 1991).

Previous research suggests that the closed-ended format triggers a different response mode in participants than the open-ended format and participants draw on different memory or reasoning processes to answer closed- and open-ended questions (Connor Desai & Reimers, 2019; Schwarz et al., 1985). Anchoring effects and a stronger tendency to follow social norms in the case of closed-ended answers may explain the differences between answers to closed- and open-ended questions (Frew et al., 2003). In some studies, both formats gave broadly similar results, but open-ended responses were more detailed (e.g., Connor Desai & Reimers, 2019). In others, closed- and open-ended questions yielded different evaluations and different justifications for these evaluations (Frew et al., 2003). Prior results are therefore mixed.

We expect that when people’s opinions are ambivalent (as can occur with attitudes towards outgroups) or when the studied phenomena are complex and people’s opinions not well-formed, there might be differences between answers from closed- and open-ended questions. If, for instance, one wants to say “it depends,” then in an open-ended question they have a chance to do so, and in a closed-ended question they might adjust to the norm in their social context. The conclusions drawn from different types of questions could be more accurate and policies based on them might better reflect people’s attitudes than conclusions from only one type of questions. Furthermore, potential interventions or programs aimed at improving the attitudes towards outgroups might better target the right aspect of these attitudes and thus might be more effective.

Recently, the interest in open-ended questions has increased in different disciplines, mainly thanks to the development of tools for automated text analysis (e.g., Baburajan et al., 2022; Connor Desai & Reimers, 2019). There are a variety of tools for automated analysis of natural language, and there is also literature on these tools and examples of research using them. Other researchers have also written more general overviews about the approaches to and methods for analyzing language in behavioral sciences (e.g., Boyd & Schwartz, 2020; Rafaeli et al., 2019). However, we find that an empirical comparison of different ways of measuring attitudes towards outgroups and a comparison of the text analysis tools conducted on the same material is lacking.

## Study context: Refugees in Poland

The goal of the current study was to compare different methods of assessing attitudes towards outgroup members and different methods of analyzing the acquired answers. To collect responses, we chose a socially important topic that evokes a variety of emotions in many countries: refugees. In 2018, according to the United Nations High Commissioner for Refugees (UNHCR), almost 70 million people worldwide were forcibly displaced, making it the highest number since the Second World War. At the moment of submitting this article (spring 2022), European countries are receiving Ukrainian refugees fleeing from their country after it was attacked by Russia. Reactions to the refugees and ideas of how they should be treated differ between countries. On the one hand, Germany opened its borders and already accepted about a million refugees from the Middle East in 2015 and 2016. On the other hand, Poland, Hungary, and the Czech Republic declared at that time that they would not accept any refugees. This strategy still holds today for the refugees from the Middle East who try to cross the border from Belarus into Poland, while the refugees from Ukraine arrive without major obstacles. In fact, in spring 2022 Poland has accepted about two million refugees from Ukraine just within a period of two weeks.

Before spring 2022, Poland had hosted only a handful of refugees. Direct contact with them was very rare. In 2017, 94% of Poles declared that they did not know any refugee personally (Stefaniak et al., 2017). Poles were rather welcoming to refugees in the spring of 2015, with 72% wanting to accept refugees in Poland. The same year, the refugees became a political topic in the parliamentary election campaign (Solska, 2017). These attitudes quickly shifted, and one year later, in the spring of 2016, only 33% of respondents wanted to accept refugees according to a Centre for Public Opinion Research (CBOS; https://www.cbos.pl/EN/about_us/about_us.php) poll (CBOS, 2018), or 27% according to an Ariadna (https://panelariadna.com/) national poll (Maison & Jasińska, 2017).

Although the above polls show overall negative attitudes towards refugees and although the Polish government has opposed admitting any to Poland until very recently, some studies suggested that the attitudes might be more complex and, if assessed in a different way, might not be as negative. A study that presented different profiles of refugees showed that “the vast majority of respondents in all surveyed countries neither categorically rejected nor categorically accepted all of their asylum-seeker profiles” (Bansak et al., 2016, p. 221). Poland fell approximately in the middle, with 45% of respondents accepting refugees, and an acceptance rate ranging between 40 and 55% in the 15 studied countries.

A question arises as to why there are such different results for the same country in a similar period of time in different surveys. All of the surveys relied on closed-ended questions, but they were formulated in a slightly different way. As there were almost no refugees in Poland before spring 2022, the vast majority of Poles have never had contact with them (Stefaniak et al., 2017). Therefore, they might have been easily influenced by the way the questions were formulated. It might also be that Poles conditioned their support for refugees on the basis of their specific attributes (e.g., their religion or employability, as in Bansak et al., 2016), and this resulted in the variability of the answers.

## Measuring attitudes towards refugees with open-ended questions

One exception to measuring attitudes towards refugees with closed-ended questions that utilized a bottom-up approach is a pilot study of refugee subgroups (Kotzur et al., 2019). In this pilot study, participants nominated meaningful categories of the subgroups of refugees (Kotzur et al., 2019). This allowed the researchers to investigate the stereotype content of a range of subgroups as identified by the participants themselves.

Another example of a study of attitudes towards refugees that used open-ended questions is an Australian study that asked the participants about their feelings, thoughts, and past experiences in relation to asylum seekers. Then, the participants quantitatively rated their own previously given open-ended answers on a continuum from *negative* to *positive* (Croucamp et al., 2017). The questionnaire did not contain separate closed-ended questions, so the researchers did not compare different ways of asking about attitudes towards refugees. However, as the authors noted, the “inclusion of a selection of participant-generated items allows insight into how the attitude processes emerge” (Croucamp et al., 2017, p. 244).

In another Australian study, the questionnaire included open-ended questions about the respondents’ attitudes towards refugees in Port Augusta (Klocker, 2004). The responses were manually coded into categories. As the author pointed out, an advantage of the open-ended question was that it provided “respondents with the opportunity to frame the asylum debate in their own terms” (Klocker, 2004, p. 4). In this case, the closed- and open-ended questions showed a similarly negative image of asylum seekers, but the open-ended questions allowed for a better understanding of the content of this image and the reasoning behind it.

## The current research

As the aforementioned results show, letting the respondents state their opinion in their own words gives additional depth to the results. In the current research, we contrasted different data collection methods to better understand the complexity of attitudes towards refugees that may not be seen using only one of the methods. Analyzing textual data manually is time-consuming, and a large sample size understandingly can become a problem. Automated tools can help researchers with text analysis, but in order to rely on these tools, it is important to know how they compare to manual coding and to understand their advantages and limitations. The overarching aim of the current research was to aid this understanding.

In our approach, we combined the breadth and depth of information. As to breadth, we conducted two surveys on relatively large samples (ca. 250–300 participants in each) that were representative of the Polish population in terms of sex, age, and place of residence. We conducted Study 2 one year after Study 1 in order to analyze a time trend in the answers. As to depth, we asked participants to respond in their own words to the question *What strategy should Poland adopt concerning refugees who want to come to Poland?* Consequently, we acquired an extensive set of opinions that were self-formulated by participants. One way of analyzing such data is to do it manually, defining themes in a bottom-up or a top-down approach. For theme formulation, we used the bottom-up thematic coding done by two independent coders in each study. Furthermore, we tested various computerized methods of analyzing textual data. The current comparison of these methods is an empirical test of different approaches (content analysis, sentiment analysis) and programs (MEH, LIWC, Sentiment Analyzer) and is aimed at helping researchers in considering which approach(es) and tool(s) to choose.

Overall, by using closed- and open-ended responses and different methods of analysis, we show what could happen when seeing the results from only one angle and using only one of all the methods we used. Later on, we discuss how one could integrate the results of all methods, but we do not suggest that all methods should be used at the same time. We compare them, discuss the differences, and recommend using more than one.

**Fig. 1:**
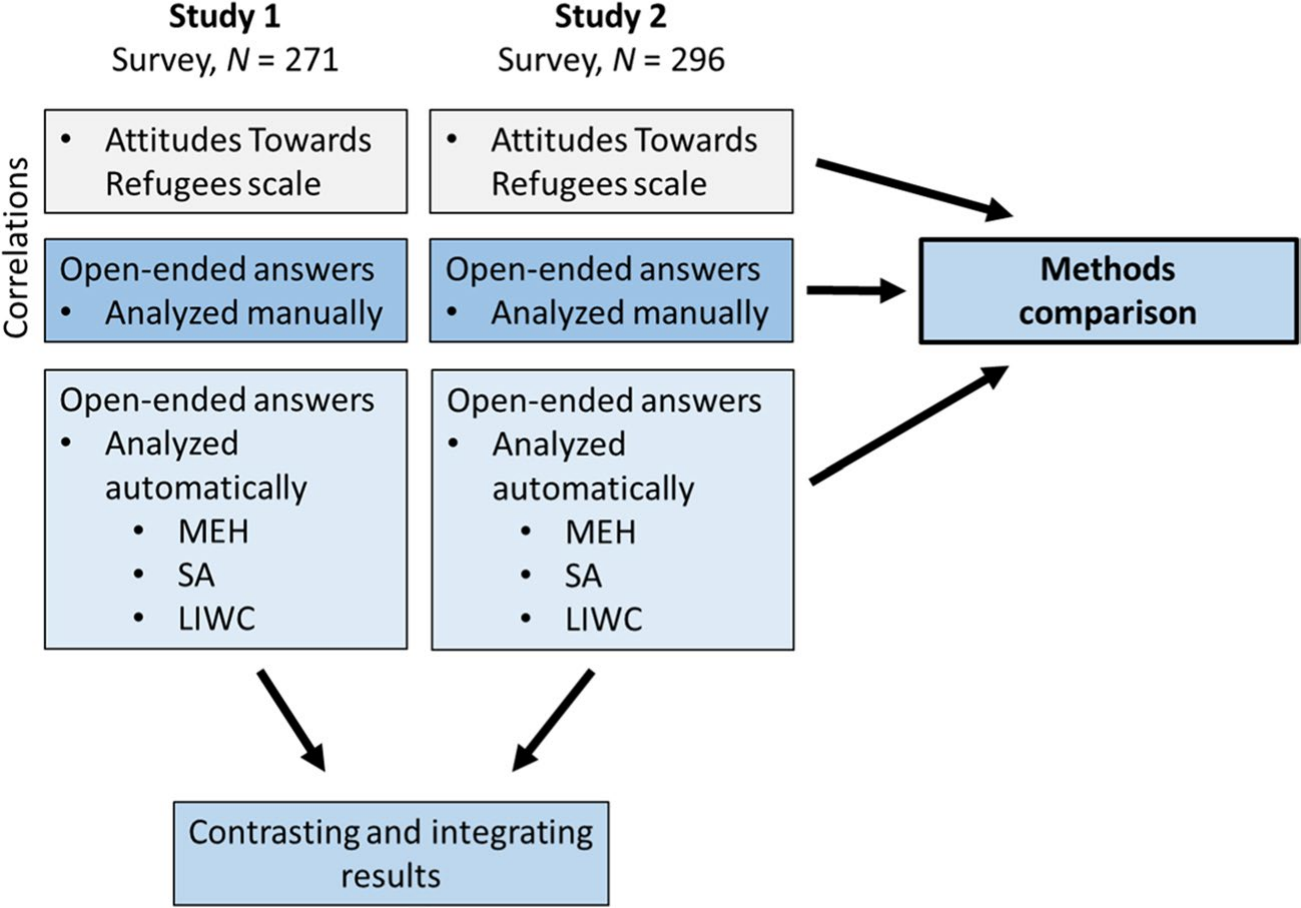
An outline of the present mixed-methods design. *Note.* MEH = Meaning Extraction Helper, SA = Sentiment Analyzer, LIWC = Linguistic Inquiry and Word Count

In the current research, we used a convergent mixed-method research design with data transformation (Creswell et al., 2003; Fetters et al., 2013). Integration of the qualitative and quantitative data took place at the data collection stage, during the analysis phase, and during the interpretation of the results (Fig. 1). As we wanted to compare different methods of analysis of the same textual material, we also transformed the data from qualitative to quantitative form. While such transformations have been discussed in the literature (e.g., Caracelli & Greene, 1993; Tashakkori & Teddlie, 1998), there is still limited guidance on the topic. Our work helps to develop standards of practice for such transformations and analyses.

## Methods of Studies 1 and 2

### Participants

We aimed to have at least 250 valid answers per study. According to the commercial research company that collected data, this sample size would be enough to reflect the demographics of the Polish population. We were also concerned with the feasibility of the study (financial resources) and of the manual text analysis (time and personal resources). We focused on comparing methods rather than statistical values, but for the simple statistical tests that we used, the achieved power was always above 99% (Faul et al., 2007).

Study 1 was completed online by 271 participants (53% women, 47% men)^1^, aged between 19 and 74 years (*M* = 43.67, *SD* = 15.11). Study 2 was completed online by 296 participants (54% women, 46% men), aged between 18 and 75 years (*M* = 43.48, *SD* = 15.68). All of them were Poles, and both samples were representative of the Polish adult population in terms of sex, age, and place of residence. The samples were collected using the computer-assisted web interviewing (CAWI) method. These were nationwide random-quota samples selected according to the representation in the population on the variables sex (2 categories) × age (5 categories) × size of place of residence (5 categories), i.e., in 50 strata in total. Table 1 presents the demographics of the Polish population as well as the demographics of our samples.^2^ Participants received compensation in accordance with the company’s terms (points that could be exchanged for prizes). Both studies were approved by the Ethics Committee of the Faculty of Psychology, University of Warsaw.

**Table 1:** Demographics (sex, age, residence) of the Polish population and of our samples

	Population	Study 1	Study 2
Women	52%	53%	54%
Men	48%	47%	46%
18–24	12%	14%	14%
25–34	20%	21%	20%
35–44	17%	16%	17%
45–54	17%	19%	18%
55+	34%	30%	31%
Rural area	38%	36%	33%
Town up to 20,000	13%	14%	13%
Town/city 20,000–99,000	20%	19%	20%
City 100,000–500,000	17%	19%	19%
City 500,000+	12%	13%	15%

### Procedure and measures

After giving their informed consent and answering basic demographic questions (used to make the samples’ structure representative), within two larger survey studies^3^, participants responded to an open-ended question: *What strategy, in your opinion, should Poland adopt concerning refugees who want to come to Poland?*

Following the open-ended question, we assessed the respondents’ attitudes towards refugees with a five-item *Attitudes Towards Refugees Scale* (α_Study1_ = .97, α_Study2_ = .94), adapted from Eisnecker and Schupp (2016). The scale included five items, four starting with *Do you think that the arrival of refugees to Poland would …* (1) *be good or bad for the Polish economy?* (response scale: *definitely bad* to *definitely good*), (2) *enrich or threaten the cultural life in Poland?* (*definitely threaten* to *definitely enrich*)*,* (3) *make Poland a better or a worse country to live in?* (*definitely worse* to *definitely better*)*, and* (4) *bring more opportunities or risks?* (*definitely more risks* to *definitely more opportunities*). The fifth item asked, *Do you think that Poland should accept some of the refugees coming to Europe?* (*definitely not* to *definitely yes*). The corresponding response scales ranged from 1 to 100 in Study 1 and from 1 to 5 in Study 2, whereby lower numbers indicated more negative attitudes. Only the endpoints of the scales were labeled. We used the mean ratings of the five items of the scale as a dependent variable.

### Codebook

The development of the codebook for the manual coding of responses to the open-ended questions followed a bottom-up approach to the analysis of qualitative data (Creswell & Poth, 2016). That is, we began with multiple rounds of reading the responses to familiarize ourselves with the data. Afterwards, we started taking notes while reading to write down our initial impressions about participants’ attitudes towards refugees. The individual work was followed by a joint meeting devoted to a discussion of data, aided by the prepared notes. We first agreed that on the most general level, participants’ responses conveyed whether they were for or against accepting refugees. Therefore, the first step of coding (to be performed later by two independent coders in each study) became to determine whether the response’s author was overall (a) *supportive of* or (b) *opposed to* accepting refugees into the country. Two additional coding options were available for answers that (c) expressed a lack of any ideas on the matter (e.g., answers such as *I don’t know*) or (d) appeared *impossible to classify* as being for or against accepting refugees (see online materials under the Open Science Framework [OSF] link and Table 3 below). At this stage, the coding was to resemble marking an answer on a scale with four (a, b, c, d) response options, where a given response can be assigned only a single code.

Further discussion about the data was focused on themes that seemed to frequently come up in the responses. We then decided that the coders should also code what recommendations participants had for the refugees themselves and/or for the receiving country. The codebook specified that the coders would mark *0* when a given theme was not mentioned in the text and *1* or *2* if it was. For most themes, only 0–1 coding was foreseen, but for some, we differentiated between different levels of the perceived strength of the answer with 0-1-2 coding. Here, any configuration of codes was possible—from all marked to none marked. This tentative plan was tested in the training phase of coding, when two coders (different people in the two studies) coded the first 10% of the responses and thus assessed the suitability of the codebook. Minor modifications were introduced based on the coders’ feedback. In the end, in Study 1 the answers opposed to the refugees could be classified into three subcategories (*refugees should be sent back home or to other countries, refugees should stay in their homeland and fight*, and *we should help Poles in need first*). The supportive answers could be divided into six subcategories of strategies. One denoted a general approval for various forms of *assistance for refugees* and the remaining five focused on approval under certain conditions: 1 = *refugees should assimilate* or 2 = *be forced to assimilate*; *refugees should be controlled by the state*; *refugees should be isolated* 1= *from society* or 2 = *from each other*; *refugees should* 1 = *work* or 2 = *be forced to work*; *refugees should* 1 = *not receive any social benefits* or 2 = *only minimal benefits*.

In Study 2, we went through a similar process of codebook development, but as a basis we used both the data and the codebook developed in Study 1. That is, while reading the responses and taking notes on them, we were checking whether the data seemed to preliminarily match the codebook or not, as well as what could be different. This led us to keep most of the categories from Study 1 and to add a few new themes. We included one new subcategory to the opposing strategies: *we should help refugees in their countries*. We also created two new subcategories for the supportive strategies: *we should fulfill international agreements* and *we should accept only certain types of people*.

### Content analysis

The coders worked independently, treating every answer as a single unit of analysis. As sometimes the replies were highly complex or even internally contradictory, the coders could assign them to multiple subcategories simultaneously. In both studies, the coders started with coding 10% of the responses in order to ascertain that the codebook is a good match for the data and to practice using it. Afterwards, the coders met to discuss discrepancies, reach agreement, and clarify potential differences in their understanding of the categories. After the training stage, minor adjustments were introduced to the subcategories in the codebook to avoid further differences in understanding and to better reflect the content of the responses. Then, the coders coded the rest of the answers. At the end, the coders met again to arrive at final decisions where disagreements still emerged. We assessed the coders’ reliability after the training stage and for the main part of coding via computation of intraclass correlation coefficients (i.e., the absolute agreement). The results showed that in Study 1, in the training phase the coders reached reliabilities of α = .96, 95% CI [.92, .98] for the primary categories, and α = .64, 95% CI [.43, .81] for the secondary categories. For the main coding (after training), the reliabilities were in Study 1 α = 1, 95% CI [1, 1] for the primary categories, and α = .60, 95% CI [.52, .67] for the secondary categories. In Study 2, in the training phase the reliabilities were α = .95, 95% CI [.90, .98] for the primary categories, and α = .52, 95% CI [.28, .72] for the secondary categories. In the main coding phase in Study 2, the reliabilities were α = .87, 95% CI [.83, .90] and α = .68, 95% CI [.62, .73], respectively. Overall, the reliabilities were high to very high for the primary categories and noticeably lower for the secondary categories. However, in the secondary categories, some codes were less prevalent and one or two disagreements could strongly influence the reliability.

### Automated text analyses

Besides the manual coding, we used three tools for automated text analysis. Each of them has advantages and limitations, and our goal was to test them on the same material and contrast their results. In future research, it might not be time-efficient to use all of them, but here, we wanted to present a practical comparison for other researchers, who can then decide which of the methods best fits their research.

#### Meaning Extraction Helper (MEH)

MEH is a tool that is used for the meaning extraction method (Boyd, 2019; Chung & Pennebaker, 2008). It uses automated text analysis to identify the most commonly used words in a text and determines how these words co-occur. Users can set the minimum number of words required for a text to be included in the analysis and the minimum observed percentage of a word (Boyd, 2019). The main MEH process occurs in three steps (Blackburn et al., 2018). First, the program automatically filters out a group of stop words (i.e., function words, low base rate words). Second, it identifies common content words (nouns, verbs, adjectives, and adverbs) in each text. Common content words are identified based on their frequency across the entire corpus that is being analyzed. MEH then assigns a binary score to each word. For example, if 10 common content words from the whole corpus are identified in a given text, a “1” will be assigned to each word and the remaining words will be assigned a “0”. In other words, the MEH generates a series of binary scores that represent common words for each text. Third, once the MEH has processed each word in each text, an output file is generated that identifies common words and shows which texts include them (or put differently, it shows each text as a row and indicates which words presented as columns are present or absent in it). Then, next steps of meaning extraction are performed outside of the MEH. The output file can be read into a statistical program (e.g., SPSS) to perform a principal component analysis (PCA) with varimax rotation and compute a set of components that identify common themes in the texts used. Based on this analysis, one can extract themes that emerge from the analyzed texts. Then, researchers can name the components using a bottom-up approach. Given the combination of statistical methods with qualitative interpretation of the components, the meaning extraction method constitutes a mixed-methods approach to studying language data. This methodology and the MEH software are recommended when conducting research in languages other than English, as the method does not involve translation until after the analyses have been conducted, which can help in cross-culturally appropriate text analysis (Ramirez-Esparza et al., 2008; Wagner et al., 2014).

#### Sentiment Analyzer

We manually corrected all responses for spelling and major grammatical errors. Subsequently, we used a tool available for Polish language called Sentiment Analyzer, part of the Common Language Resources and Technology Infrastructure, available online at https://ws.clarin-pl.eu/sentyment^4^. The tool’s development drew on a lexical semantic network for Polish, i.e., plWordNet 3.0 (Maziarz et al., 2013), which became one of the largest Polish dictionaries (Janz et al., 2017). plWordNet comprises lexical units (i.e., lemma, part of speech, and sense identifier, which together constitute a lexical meaning) and to a subset of these units, emotive annotations were added manually (Zaśko-Zielińska et al., 2015). In short, the annotators first identified the sentiment polarity of the lexical units (positive, negative, and neutral). Second, they assigned basic emotions following Plutchik’s (1980) wheel of emotions (joy, sadness, anger, fear, disgust, trust, and anticipation). Moreover, in the Polish linguistic tradition, basic emotions are associated with fundamental human values (Zaśko-Zielińska et al., 2015), and it may be difficult to separate emotions from values in language expression (Kaproń-Charzyńska, 2014). Therefore, six positive (utility, another’s good, beauty, truth, happiness, knowledge) and six negative (ugliness, error, harm, misfortune, futility, ignorance; Puzynina, 1992) values were incorporated into the unit descriptions.

#### Linguistic Inquiry and Word Count (LIWC)

To be able to analyze the responses in the LIWC program (Pennebaker et al., 2015), we translated them from Polish to English via Google Translate (https://translate.google.pl/). Such approach was recommended to us by the LIWC developers for datasets in languages not covered by the software, and it has been shown to be effective in other studies (R. Boyd, personal communication, June 21, 2018). LIWC consists of the processing component that opens text files and the dictionaries. The program goes through each text in a file, word by word, and compares each word with the dictionary file, which consists of nearly 6400 units (words, word stems, and emoticons). If the word appears in the dictionary, it is automatically counted and classified into hierarchically-organized categories. At the end, LIWC calculates percentages of the categories. The categories include 21 linguistic dimensions (e.g., pronouns, verbs), 41 psychological constructs (e.g., perception, affect, and cognition), six personal concerns (e.g., work, home), and five informal language markers (e.g., swear words). In addition, LIWC provides the word count, general descriptor categories (e.g., words per sentence), and summary language variables (e.g., emotional tone; Pennebaker et al., 2015).

#### Automated text analyses: Short methods comparison

MEH allows researchers to automatically extract themes that emerge from open-ended answers. Its advantage is that it gives a quick impression of these themes and is language-independent, but the downside is that it does not take valence or negation into account. In sum, it can identify topics, but not emotions. Sentiment Analyzer allows its users to analyze valence, emotions, and associated values in the responses, and it is tailored for the Polish language, which is more grammatically complex than English. LIWC is to some extent similar to Sentiment Analyzer, but there are many more categories in LIWC. The downside is that LIWC is language-dependent and while it is available in a few languages, there is no official version for Polish. Additionally, both of the dictionary-based programs, Sentiment Analyzer and LIWC, have little or no capacity to account for context, irony and sarcasm, or idioms (Tausczik & Pennebaker, 2010). By using all these methods on the same material, we present their advantages and disadvantages in practice.

## Results of Studies 1 and 2

### Attitudes Towards Refugees Scale

The attitudes towards refugees were measured with five closed-ended statements of the *Attitudes Towards Refugees Scale*. They reached *M* = 38.42 (*SD* = 28.62) on a response scale from 1 to 100 in Study 1, and *M* = 2.33 (*SD* = 1.20) on a response scale from 1 to 5 in Study 2, where lower levels meant more negative attitudes. In both time points the results of one-sample *t*-tests showed that the means were significantly lower than the scales’ midpoints (38.42 vs. 50.5 in Study 1, and 2.33 vs. 3 in Study 2), with *t*(270) = −6.95, *p < *.001, 95% CI [−15.50, −8.66], Cohen’s *d* = −0.42, for Study 1, and *t*(295) = −9.55, *p < *.001, 95% CI [−0.81, −0.53], Cohen’s *d* = −0.56, for Study 2.

**Fig. 2:**
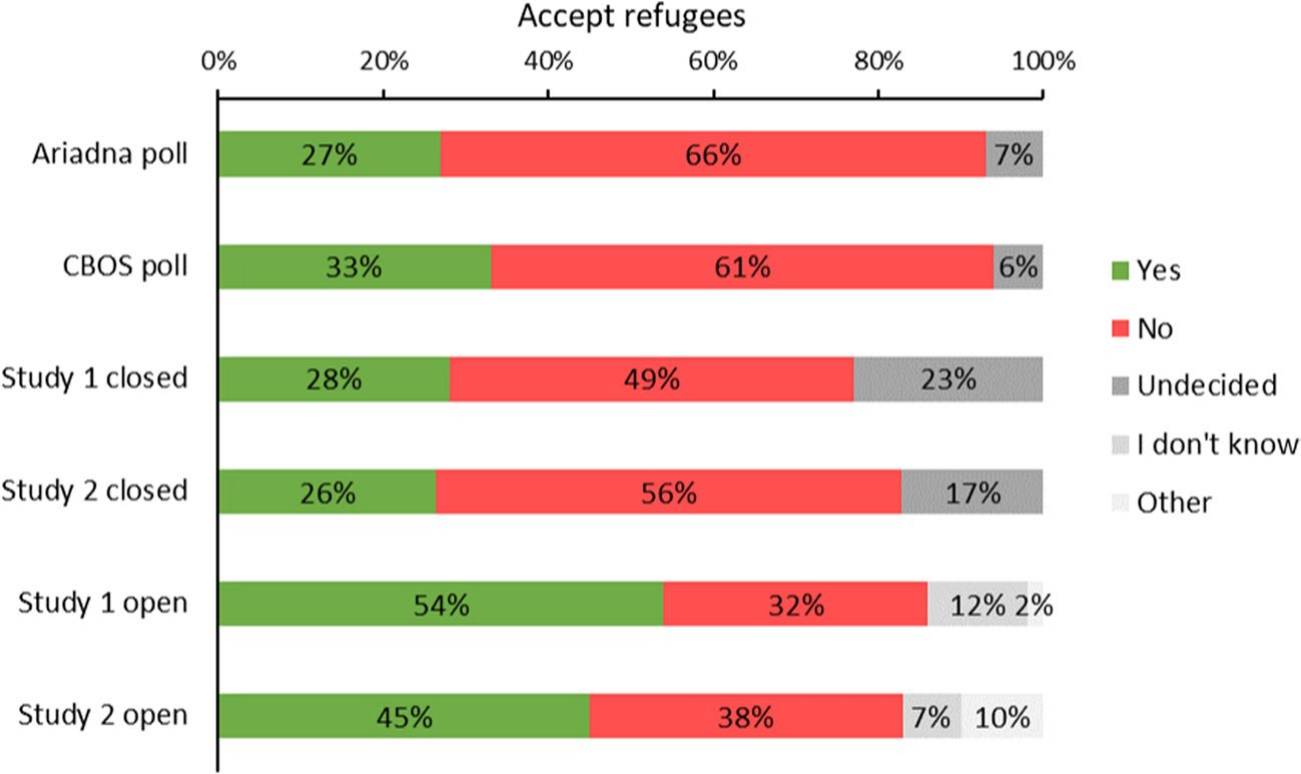
Results of two national polls, closed-ended answers from Studies 1 and 2, and manual coding of open-ended answers in Studies 1 and 2

In order to compare the responses to the scale across our Studies 1 and 2, but also to compare it to studies of national polls (CBOS and Ariadna) conducted at a similar time, we concentrated on the question *Do you think that Poland should accept some of the refugees coming to Europe?* This question was very similar to questions asked in these polls and had the same anchors of the response scale (*definitely no* to *definitely yes*). We first recoded the 1–100 variable from Study 1 into a five-point scale (1–20 → 1, 21–40 → 2, 41–60 → 3, 61–80 → 4, 81–100 → 5). Then, in the same manner for Studies 1 and 2, we combined frequencies for responses of 4 and 5 into “supportive of accepting refugees” and frequencies for responses of 1 and 2 into “opposed,” and 3—the midpoint of the scale—we treated as “undecided.” When looking at the percentages in Study 1, 28% of the participants were supportive, 49% were opposed, and 23% were undecided (see Fig. 2). In Study 2, 26% of the participants were supportive, 56% were opposed, and 17% were undecided (see Fig. 2). The above results suggest that the participants’ attitudes expressed via the closed-ended statement were generally negative and were in opposition to allowing refugees in Poland. This attitude did not change significantly across Studies 1 and 2, as evidenced by the results of an analysis of variance (ANOVA) with Study (1 vs. 2) as the between-subjects factor and the mean responses to the aforementioned question from the scale as the dependent variable, *F*(1, 565) = 1.55, *p = *.39, η_p_^2^ = .00 (*M*_*Study1*_ = 2.56, *SD* = 1.41 vs. *M*_*Study2*_ = 2.45, *SD* = 1.49).

### Manual content analysis

The open-ended answers that we analyzed via the manual coding as well as with automated text analyses were of varying length (Table 2): some responses were very short (one to a few words) and some were very long (a paragraph), but most comprised one or two sentences.

**Table 2:** Corpora descriptives

	Minimum word number	Maximum word number	Mean	SD
Study 1	1	183	16.64	22.68
Study 2	1	199	14.99	22.72

As mentioned in the Methods section, in the manual content analysis of the answers to the open-ended question, there were two levels of codes: a primary code/category (*accept, do not accept, I don’t know,* and *other*), which all answers were ascribed one of, and a secondary code/category (e.g., *send refugees home or to other countries*), any number of which was assigned to any given answer. The results of the content analysis revealed that in Study 1, 54% of the participants were supportive of accepting refugees and 32% were opposed (see Fig. 2). In Study 2, 45% of participants were supportive and 38% were opposed. The rest of the participants were undecided (*I don’t know* answers: 12% in Study 1 and 7% in Study 2) or gave answers that were impossible to code as being supportive or opposed (2% and 10%, respectively).^5^ The general categories were subdivided into more specific themes, as it was important for us to not only interpret the answers quantitatively in terms of percentages for and against, but also to examine their qualitative content (see Table 3). The themes were thus nested under the primary categories (e.g., *refugees should assimilate* was a subcategory of *accept*). We report the prevalence percentages out of all answers, not only out of the given primary category.

**Table 3:** Results of the manual content analysis in Studies 1 and 2, as well as (whole or fragments) of two example comments

Category label	Study 1	Study 2	Two example comments or their fragments
Do not accept refugees	32%	38%	*1. Do not accept.* *2. Definitely do not accept them to Poland.*
Send refugees home or to other countries	18%	-	*1. Send them back.* *2. They should be placed in separate places and sent further.*
Refugees should stay in their homeland and fight	3%	1%	*1. They should go back to their country and fight.* *2. Poland should only accept mothers with children. Men should stay in Syria and fight for the freedom of their homeland.*
We should help Poles in need first	4%	3%	*1. In Poland, there live very poor people and we should help them and not accept others.* *2. We are not a country that would be rich enough to additionally accept refugees*
Help refugees in their countries	-	9%	*1. Poland should support refugees in their home countries.* *2. [Poland] should direct help to Syria.*
Accept refugees	54%	45%	*1. Let them in, give them work.* *2. Accept refugees and integrate them.*
Provide support	23%	7%	*1. Care for them, help them.* *2. We should let some refugees into the country and help them assimilate here in Poland.*
Refugees should assimilate including being forced to assimilate	22% 6%	6% 2%	*1. Allocate them, teach them the language and customs, give the opportunity to earn money.* *2. Accept, people who want to stay should be 100% assimilated with Polish society.*
Refugees should be controlled by the state	12%	15%	*1. Strict control of them.* *2. Thorough control, and not creating refugee clusters.*
Refugees should be isolated from society or from each other	10%	2%	*1. Separate them into groups and allocate as far away as possible.* *2. Accept refugees and keep them in refugee centers until assimilation and thorough verification of their identity.*
Refugees should work including being forced to work	30% 16%	5% 1%	*1. They should be admitted to our country, get help in finding a job and be enabled to integrate with the local community * *2. Constant supervision and continuous work for the benefit of the Polish economy.*
Refugees should not receive many social benefits or any social benefits	12%	2%	*1. They should adapt to Polish realities and start working, not live on benefits.* *2. Rigorous checks, obligation of any kind of employment for a specific period of time instead of getting social benefits.*
We should fulfill international agreements	-	3%	*1. We should fulfill international agreements.* *2. [Poland] should accept as many refugees as ordered by the EU.*
We should accept only a certain number or a certain kind of people	-	25%	*1. If accept, then Christians and no single guys.* *2. Accept the 7000 refugees promised by the previous government, women with children.*
I don’t know	12%	7%	*1. I don’t know.* *2. I have no opinion.*
Other (impossible to classify as for or against)	2%	10%	*1. That what should be done.* *2. The question is a little absurd because there are no such people...*

The four main categories (*accept, do not accept, I don’t know*, and *other*) sum up to 100%

Most of the answers stated that refugees should be accepted, but only 23% of all the answers in Study 1 and 7% in Study 2 explicitly described the support that the refugees should receive. The most frequent topic brought up in Study 1 was that refugees should work—30% of participants mentioned it, and 16% even said that refugees in Poland should be forced to work (see Table 3). The second most frequent category was to assist refugees in a variety of ways (23%). The third was a recommendation that they should assimilate into Polish society (22% mentioned assimilation, and 6% said refugees should be forced to assimilate). These themes did not surface to a similar extent in Study 2. Instead, the participants in Study 2 focused on the need to carefully select those who were to arrive (25%) and on the necessity for the authorities to control them (15%, see Table 3). In both time points, many of the “accept” answers were rather of the “yes, but…” kind and stated what refugees should do or under what conditions they should be accepted (see examples of comments fragments in Table 3).

Among people who were opposed to accepting refugees in Study 1, most said that Poland should send refugees back home or to other countries, while others indicated that refugees should have stayed in their homeland and fought. In Study 2, conducted a year later, an idea emerged that Poland should help the refugees in their home countries. This may be related to the fact that such an answer to the refugee situation was at that time mentioned in the media (e.g., Polsat News, 2017; TVN24, 2017).

### First integration: Attitudes scale and manual coding

We wanted to triangulate and integrate the results from the closed- and open-ended answers. To this end, we followed the approach recommended in the literature (e.g., Onwuegbuzie & Teddlie, 2003) and we correlated the answers from the closed-ended questions with the codes stemming from the open-ended questions. We also added another analysis: mean comparisons using a series of *t*-tests.^6^ For the correlations, we binarized a few codes that had a 0-1-2 coding into a 0-1 coding. All variables from the manual coding that were usable were included. From the first variable, we included only 0 (*do not accept refugees*) and 1 (*accept refugees*), without *I don’t know* and *other*. Then, we took each of the codes as a separate variable (0 = code absent, 1 = code present) and we ran Pearson’s correlations on each of these code variables with mean scores on the *Attitudes Towards Refugees Scale* (scale: 1–100 or 1–5). For the mean comparisons, we computed *t*-tests with codes treated as groups (i.e., we compared *code absent* vs. *code present* groups) with the *Attitudes* scale as a dependent variable. Having two studies, we could verify the results from Study 1 on the data from Study 2. Even though the frequencies of the themes shifted between Study 1 and 2, the results of the relationships between these measures as well as the results of the mean comparisons were similar in both studies.

**Fig. 3:**
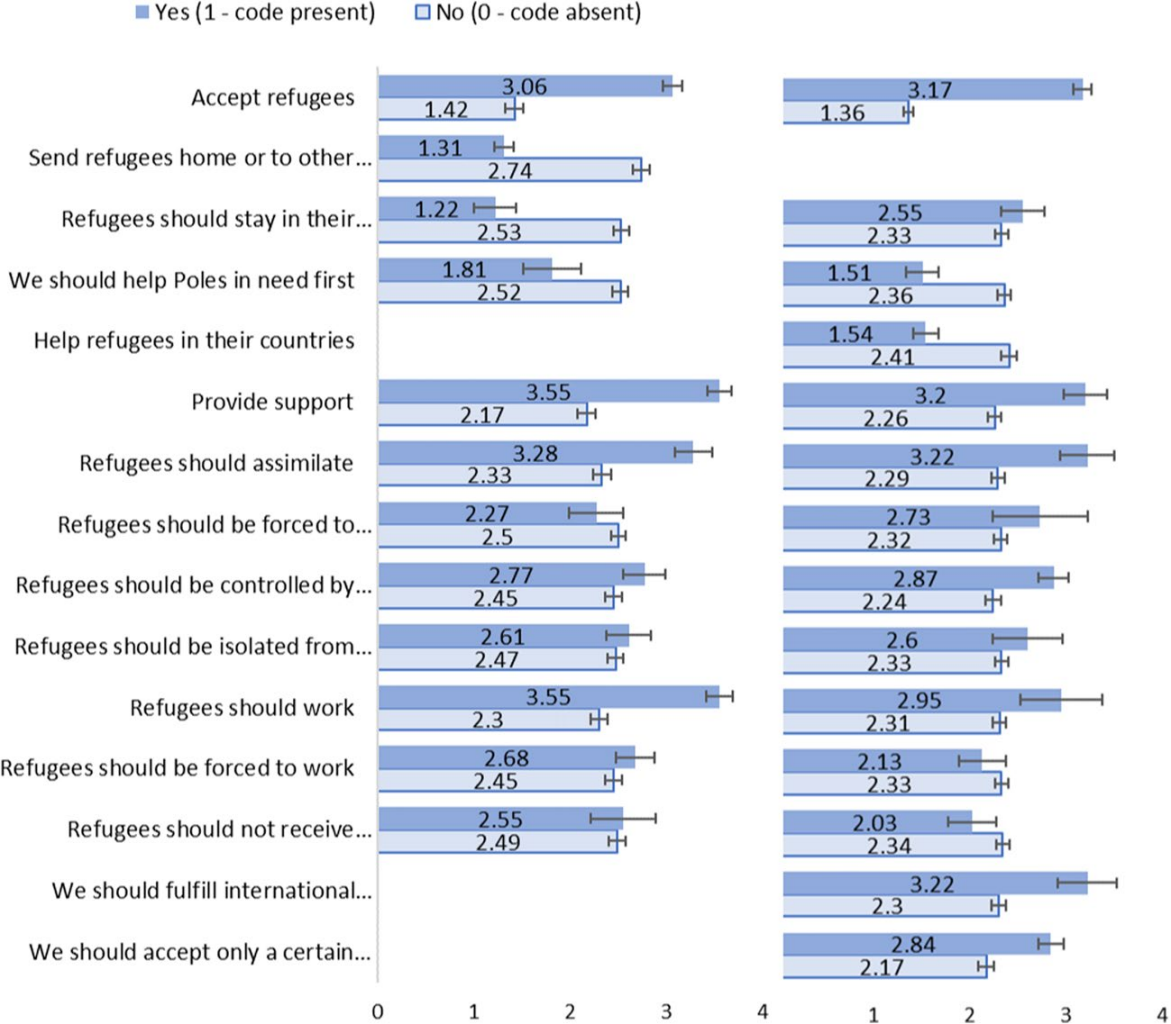
Mean differences in the Attitudes Towards Refugees Scale between code present and code absent in the results of the manual coding in Studies 1 and 2. *Note.* Error bars represent standard errors of the mean

The correlations showed that the results from the scale were strongly correlated with the results coded as the *Accept refugees* category (Table 4, left). The mean comparisons showed clearly that people who said that refugees should be accepted were also on the *Attitudes* scale more welcoming than people who said that refugees should not be accepted (Fig. 3). The Cohen’s *d* value showed that the effect was very large (Table 4, right). The correlations and mean comparisons also reflected the ambiguity of the answers that was visible in the manual coding. For example, mentioning that the refugees should assimilate (e.g., learn the Polish language) was correlated with positive attitudes towards them and people saying it had more positive attitudes towards refugees than people not mentioning it. However, saying that refugees should be forced to assimilate was not correlated with attitudes and there were no significant differences in attitudes.

**Table 4:** Correlations between the Attitudes Towards Refugees Scale and the results of the manual coding and Cohen’s d for mean difference in these attitudes between code present and code absent in Studies 1 and 2

Manual coding	Correlations with attitudes		Cohen’s *d* for mean difference	
	Study 1	Study 2	Study 1	Study 2
Accept refugees	.60***	.73***	1.49***	2.15***
Send refugees home or to other countries	−.45***	-	−1.18***	-
Refugees should stay in their homeland and fight	−.18**	.02	−1.00***	0.18
We should help Poles in need first	−.12*	−.12*	−0.52*	−0.71***
Help refugees in their countries	-	−.21***	-	−0.74***
Provide support	.46***	.21***	1.15***	0.80***
Refugees should assimilate	.29***	.15**	0.74***	0.77**
Refugees should be forced to assimilate	−.03	.05	−0.17	0.34
Refugees should be controlled by the state	.06	.18**	0.24	0.53***
Refugees should be isolated from society or from each other	.02	.03	0.10	0.23
Refugees should work	.34***	.10	0.99***	0.53*
Refugees should be forced to work	.08	−.02	0.18	−0.17
Refugees should not receive much social benefits or any social benefits	−.01	−.04	0.04	−0.25
We should fulfill international agreements	-	.13*	-	0.77*
We should accept only a certain kind of people	-	.24***	-	0.57***

*p < .05, **p < .01, ***p < .001

Some correlations and mean differences were unsurprising, for example, that people who thought the refugees should be sent home had much less positive attitudes towards them. Some results were, nevertheless, intriguing considering the up-to-now understanding of attitudes towards refugees based on closed-ended answers, but less so given our results of the open-ended questions. For instance, mentioning that refugees should assimilate or should work, or that Poland should accept only a certain kind of people does not sound like an expression of positive attitudes towards refugees at first. However, letting participants express their opinion in their own words gave us insight into why these were associated with positive attitudes. People who indicated on the closed-ended questions that refugees could be enriching for the country and that we should accept them were often reasoning in their answers that refugees who will be well-integrated, will work, and will be deemed harmless could indeed enrich Poland and its economy and should be accepted.

### MEH

As the next step of our analyses, we compared the manual coding of the open-ended answers with the results from MEH, an automated tool designed to extract themes from textual data. We set the minimum number of words required for a text to be included in the analysis to 2, and the minimum observed percentage to 3% (see Boyd, 2019; Ikizer et al., 2019). We conducted a PCA with a varimax rotation for each of the two studies. The variables entered were the content words that appeared in the texts. They were automatically coded for whether they were present in a given text or not (for more details, see Tables 2 and 3 in the Supplementary Material on OSF). As PCAs on textual data can produce too many components, it is advisable to use a higher eigenvalue than the customary 1 for determining the number of factors. We used an eigenvalue of ≥ 1.5.

The results showed that four themes in Study 1 were above the eigenvalue of ≥ 1.5. These themes explained 29% of variance, which is fully acceptable with this type of data (Boyd, 2019). There were eight themes above the eigenvalue of ≥ 1.5 in Study 2 that explained 44% of variance (for more details see the Supplementary Material on OSF). Each answer was quantified for the degree to which it fit (i.e., loaded on) each of the themes. In order to name the themes, we analyzed the words in the themes and sample texts that fit each of the themes the best. Table 5 presents the theme labels, example words, and (fragments of) the two highest-loading comments in each theme.

**Table 5:** Extracted themes, example words, and fragments of first two highest-loading comments in each theme in Studies 1 and 2

Study 1			Study 2		
Theme label	Example words	Fragments of first two comments	Theme label	Example words	Fragments of first two comments
Support and work	Apartment, work, possibility, provide, give	*1. They should be guaranteed flats, places at school, and work.* *2. Have apartments provided, have the opportunity to work.*	Support and assimilation	Should, good, country, financial, let in	*1. We should help refugees. But not to let them in into the country. Help financially, send humanitarian aid.* *2. We should let a number of refugees into the country and help them assimilate here in Poland.*
Forced work	Social benefits, let/should, only, want, work, refugee, accept	*1. They should abandon their traditions and habits and follow the same principles as we do. They should be given a chance to work, not just live on the unemployment benefit.* *2. They should work, accept only refugees who want to work and assimilate, not to receive benefits.*	Forced work	Help/support, country, work, life, belong/should, person	*1. In my opinion, Poland can provide emergency aid to people injured in the armed conflict in Syria, but I believe that the complete opening of the borders to refugees who are not focused on work, but on social support from the state, will not be the right decision.* *2. The support of the Polish state should consist of help in finding a job and a flat, Poland should not support such people financially (social benefits), but give them the possibility of a normal life.*
Let them in but…	Country, own, place, good, go, can, should, life, accept, check	*1. I believe that Poland cannot give any numbers but accept them only in individual cases, as it has been done so far.* *2. Stay in centers for immigrants until the end of the war in their country. Christians who had actively participated in church life earlier in their country could stay permanently.*	Faking to flee from war/help abroad	Flee, war, mother, Syria	*1. As can be seen from television reports, men predominate in the crowd of refugees, young and middle-aged - they run away from the obligation to defend their country (...) The best help is to help solve problems in their countries.* *2. Poland should help refugees in their homeland. I don't understand why we have to help healthy young men who left their children, their mothers, wives, and ran away.*
Language and assimilation	Learning, language, Polish, help, culture	*1. It seems to me that if they would like to stay with us permanently, they should assimilate to us, but retaining the right to their own culture and their children should have the right to learn their mother tongue, in addition to Polish.* *2. The Syrians had food, accommodation and Polish language training (... but) they fled to Germany. The refugees do not want to go to Poland.*	Control and selection	Accept, Poland, check, bad, Christian	*1. After checking that these refugees did not participate in any training (terrorist, rebel, insurgent, etc.), did not participate in the war on either of the sides, and their home, workplace, etc. don't exist, a limited number of them can be accepted.* *2. Accept refugees after verifying their identity and signing a declaration of compliance with Polish law.*
			Women and children only	Child, woman, family, only, go/walk	*1. Accept, but only women and children.* *2. Accept mainly Syrian Christians, families with children, women with children, and give preference to educated people.*
			Polish government should/shouldn't	Should, Polish, opinion, refugee	*1. In my opinion, the people whom Poland would accept, would have to, first of all, adapt to the rules in our country.* *2. In my opinion, Poland should not accept refugees.*
			Poland - bad place and time	Time, think/be careful, can, accept/acceptance, Pole(s), have, space	*1. I am ashamed of this government and think that Poles should overthrow it first and then create a refugee admission strategy.* *2. I am against admitting refugees to Poland, but if such a situation arises, they should be under constant surveillance.*
			They don't want to come	Come, want, everything	*1. To sum up: if no one wants, neither the majority of Poles nor themselves - why force anyone to do it?* *2. First of all, will anyone even want to come here?*

**Fig. 4:**
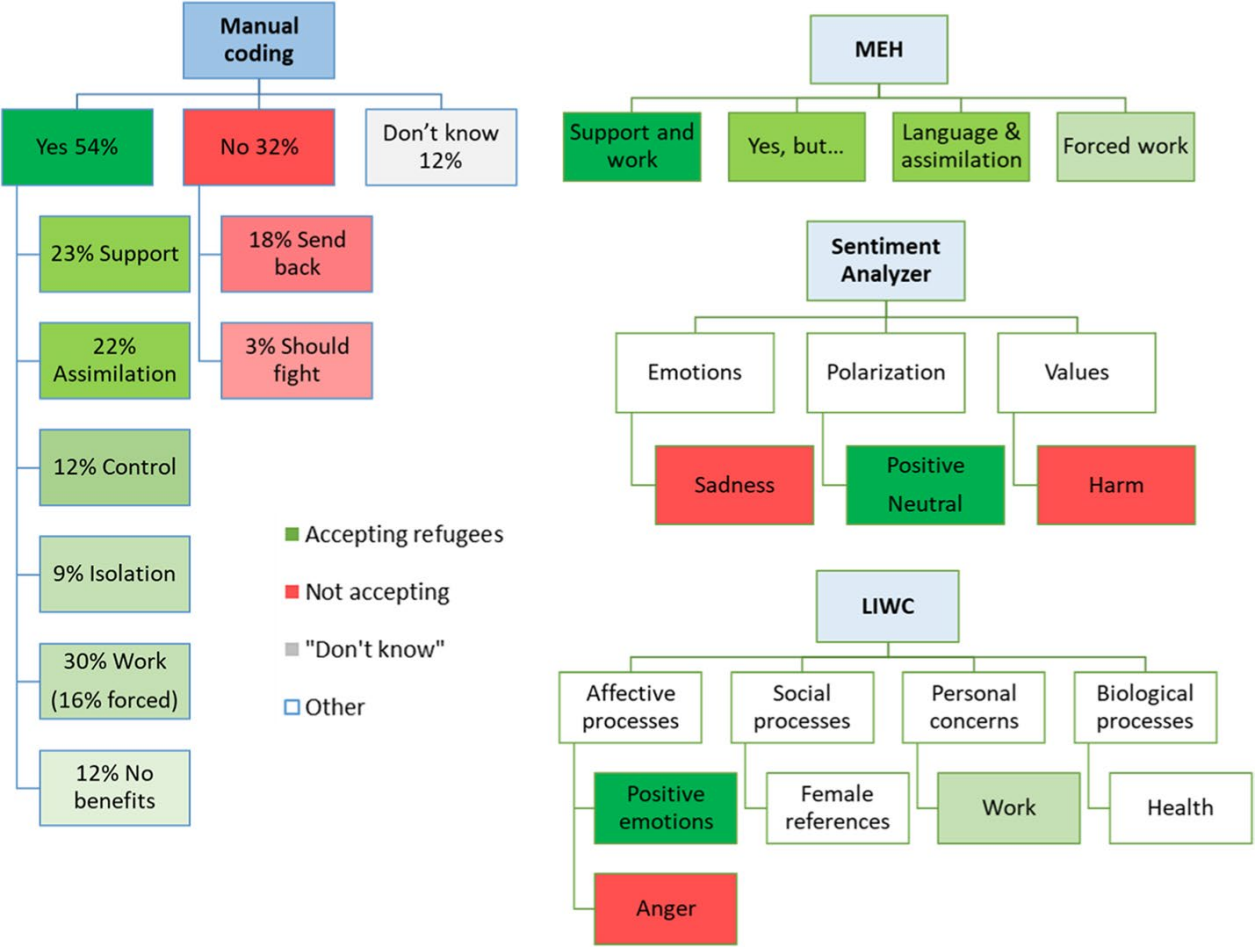
Categories that emerged from analyses of the same open-ended answers in Study 1 when using different methods. *Note.* MEH = Meaning Extraction Helper, LIWC = Linguistic Inquiry and Word Count

In Study 1, the four themes were: *Support and work*, *Forced work*, *Let them in but…*, and *Language and assimilation*. The two work-related themes reflected the work-related theme from the manual coding results (see Fig. 4), but were structured slightly differently. The first theme combined working with other types of support that the refugees should be provided. The second theme reflected the forced work theme of the manual coding but also incorporated the *No social benefits* theme. The *Let them in but…* theme was the broadest one, and it partly reflected our *Accept* category from the manual coding when subtracting the *Provide support* theme—it showed that even Poles who indicated that we should accept refugees listed many conditions under which it should happen. The *Language and assimilation* theme was partly similar to the manually coded *Assimilation* theme, but it was more focused on language only and it expressed both supportive statements about language courses that should be provided with rather unfriendly statements about the supposed lack of will of refugees to integrate and learn the local language.

In Study 2, the eight themes that emerged were *Support and assimilation, Forced work, Faking to flee from war—help abroad, Control and selection, Women and children only, Polish government should/shouldn't, Poland—bad place and time,* and *They don't want to come*. Thus, there was some overlap between the themes from Study 1 and the categories from manual coding in Study 2, but new themes also emerged. Specifically, the new themes included statements that Poland is not the best country to invite refugees to and that refugees genuinely do not want to come to Poland, but to other EU countries. However, these themes were the smallest ones, with the least variance explained (see Table 5 and the Supplementary Material on OSF). Again, even those themes that were to some extent similar to the manual coding included statements that were on the same topic but had different valence or intention.

### Sentiment Analyzer

To further explore the attitudes expressed in participants’ replies to the open-ended questions, we subjected the obtained texts to automated sentiment analysis. For this purpose, we selected a subset of variables that can be generated by Sentiment Analyzer, We chose those that also surfaced in participants’ responses in the manual coding and MEH. We considered only the responses coded as supportive of or opposed to accepting refugees into Poland, excluding “I don’t know” and other indecisive answers. Consequently, the Sentiment Analyzer variables included in the statistical analyses as the dependent variables were: polarity (positive, negative, neutral), five emotions (anticipation, fear, disgust, anger, sadness), and three values (harm, futility, utility). We conducted three multivariate analyses of variance (MANOVAs) on these three sets of variables with Study (1 vs. 2) and Response (for vs. against accepting refugees) as two between-participants factors.

The multivariate main effect of study was significant, *F*(3, 471) = 6.57, *p < *.001, η_p_^2^ = .04, and the effect of response marginally significant, *F*(3, 471) = 2.28, *p = *.079, η_p_^2^ = .01. These effects were qualified by a significant interaction, *F*(3, 471) = 2.96, *p = *.032, η_p_^2^ = .02. In the univariate tests, the interaction was significant for positive polarity, *F*(1, 473) = 4.50, *p = *.034, η_p_^2^ = .01 and neutral polarity, *F*(1, 473) = 5.81, *p = *.016, η_p_^2^ = .01. The pairwise comparisons showed, for Study 1 versus Study 2, that responses for accepting refugees were more positive in Study 2 than in Study 1 (*M*_Study1_ = 0.44, *SD* = 0.80 vs. *M*_Study2_ = 0.86, *SD* = 1.65,* p* = .002). Responses against accepting refugees were in Study 2 less neutral (i.e., stronger) than in Study 1 (*M*_Study1_ = 7.80, *SD* = 12.34 vs. *M*_Study2_ = 3.95, *SD* = 4.20, *p* = .007). Further, comparing responses for and against refugees, only in Study 2 these responses differed: responses for accepting refugees were more positive than responses against refugees (*M*_for_ = 0.86, *SD* = 1.65 vs. *M*_against_ = 0.42, *SD* = 0.94, *p* = .003). Responses for accepting refugees were also more neutral (i.e., weaker) than responses against accepting refugees (*M*_for_ = 8.54, *SD* = 11.64 vs. *M*_against_ = 3.95, *SD* = 4.20, *p* < .001).

For emotions and values, only the multivariate main effect of study was significant, for emotions, *F*(5, 469) = 2.76, *p = *.018, η_p_^2^ = .03, and for values, *F*(3, 471) = 5.40, *p = *.001, η_p_^2^ = .03. In the univariate tests, the main effect of study was significant for the emotion of sadness, *F*(1, 473) = 5.08, *p = *.025, η_p_^2^ = .01 and the value of harm, *F*(1, 473) = 7.50, *p = *.006, η_p_^2^ = .02. References to sadness appeared more in Study 2 than in Study 1 (*M*_Study1_ = .38, *SD* = 1.02 vs. *M*_Study2_ = .60, *SD* = 1.14). Similarly, references to harm appeared more in Study 2 than in Study 1 (*M*_Study1_ = .34, *SD* = .94 vs. *M*_Study2_ = .60, *SD* = 1.24).

Taken together, the polarity of words that participants used in their responses may suggest less intense attitudes towards refugees in Study 1 than in Study 2. Simplifying, one could define them as overall more positive in Study 2. The results were, however, more complex and showed rather a larger polarisation than only positivity. The neutrality and positivity results were combined with the expression of these attitudes by participants that were (based on the manual coding) in favor or against accepting refugees. This showed that participants who were in favor of accepting refugees were even more positive in their answers in Study 2 than in Study 1. However, participants in Study 2 simultaneously expressed more concerns about having refugees in Poland. These results are partially in line with the results of our content analyses in that participants’ attitudes in Study 1 were overall more positive than a year later. On the other hand, although fewer participants were for the idea of accepting refugees in Study 2, perhaps they used stronger words to convey their approval than participants in Study 1. At the same time, participants in Study 2 might have been sad about the dire situation of refugees and the harm inflicted on them, which would fit the positive attitudes. Nonetheless, from the content analyses, we know that participants did not empathize with refugees, but rather were worried about the consequences of their arrival.

### LIWC

Sentiment Analyzer was the only tool at our disposal to analyze responses in Polish without translating them. It was intended to focus on, obviously, the linguistic expressions of feelings and emotions and their valence. We subsequently turned to LIWC, which is an established tool for more extensive automated text analysis. The program generates about 90 output variables for each text file, but not all of the available LIWC categories were pertinent to the present purpose. First, we used the overall word count from the output and reported it for the two studies in Table 2. In order to determine which variables should be further analyzed in order to explore the underlying language structure of the answers to the open-ended questions, we again reviewed the results of the manual coding and MEH analyses. We compared them with examples of words, constituting LIWC categories, in the program’s dictionary (Pennebaker et al., 2015). This led us to identify affect (positive emotions, negative emotions, anxiety, anger, sadness), personal concerns (work, home, money), biological processes (body, health, power, risk), and social processes (family, male, female) as four potential categories of interest. As in the Sentiment Analyzer, the variables that make up the categories are conceptually related (e.g., Tausczik & Pennebaker, 2010; Pennebaker et al., 2015). We therefore conducted four MANOVAs (one per category) with the selected variables from each given category as the dependent variables. Study (1 vs. 2) and Response (for vs. against accepting refugees) were the between-participants factors.

The multivariate main effect of study was significant for affect, *F*(5, 469) = 3.63, *p = *.003, η_p_^2^ = .04. For affect, the multivariate main effect of response and the interaction were marginally significant, *F*(5, 469) = 1.96, *p = *.084, η_p_^2^ = .02 and *F*(5, 469) = 2.03, *p = *.074, η_p_^2^ = .02, respectively. We discuss further only the significant result. Specifically, the univariate main effect of study was significant for positive emotions, *F*(1, 471) = 10.78, *p = *.001, η_p_^2^ = .02 and anger, *F*(1, 471) = 4.41, *p = *.036, η_p_^2^ = .01. The amount of positive emotions was higher in Study 2 (*M* = 5.65, *SD* = 11.57) compared to Study 1 (*M* = 2.84, *SD* = 5.06), while anger was lower in Study 2 (*M* = .28, *SD* = 1.59) compared to Study 1 (*M* = 1.02, *SD* = 6.77).

For personal concerns, the multivariate main effects of study and response were significant, *F*(3, 469) = 10.54, *p < *.001, η_p_^2^ = .06 and *F*(3, 469) = 19.82, *p < *.001, η_p_^2^ = .11, respectively. The interaction was significant as well, *F*(3, 469) = 12.75, *p < *.001, η_p_^2^ = .08, but just for work in the univariate analysis, *F*(1, 471) = 36.19, *p < *.001, η_p_^2^ = .07. The pairwise comparisons revealed that participants who were for accepting refugees mentioned work more often in Study 1 than in Study 2 (*M*_Study1_ = 9.38, *SD* = 11.35 vs. *M*_Study2_ = 1.78, *SD* = 4.00, *p* < .001), and in Study 1 they mentioned it more than those who were against accepting refugees (*M = *.90, *SD = *2.76, *p* < .001).

For biological processes, the multivariate main effect turned out significant for response, *F*(4, 468) = 3.88, *p = *.004, η_p_^2^ = .03, and the univariate main effect of response was significant for health, *F*(1, 471) = 12.27, *p < *.001, η_p_^2^ = .03. That is, participants in favor of accepting refugees referred to health more (*M* = .97, *SD* = 3.37) than participants who were against (*M* = .09, *SD* = .52).

Finally, for social processes, the multivariate main effect of study was significant, *F*(3, 469) = 4.53, *p = *.004, η_p_^2^ = .03 and response was marginally significant, *F*(3, 469) = 2.54, *p = *.056, η_p_^2^ = .02). In terms of the univariate tests, the main effect of study was significant for the variable female, *F*(1, 471) = 13.39, *p < *.001, η_p_^2^ = .04. There were more references to females in Study 2 (*M* = 1.28, *SD* = 4.62) than in Study 1 (*M* = .11, *SD* = .74).

In sum, the results of LIWC analyses converged with those from Sentiment Analyzer with regard to a generally more positive valence of responses in Study 2 compared to Study 1. The difference was that Sentiment Analyzer did not detect anger, while LIWC did not detect sadness when it comes to particular emotions in the answers. Therefore, the results of the automated analyses of the emotional underpinnings of the responses may be deemed quite inconclusive. This said, we did not explicitly code emotions, but instead inferred them post hoc so that we could explore the data with both Sentiment Analyzer and LIWC. Considering other LIWC variables, work did surface earlier in the manual coding and MEH analysis, in the participants’ opinion that refugees should have jobs or even be forced to work. This issue was indeed, like in LIWC results, more emphasized in Study 1. We have also noticed specifications as to who may be allowed to enter Poland, and an inclination to accept female refugees in Study 2 (see e.g., Table 5). A somewhat unexpected result concerned health in the responses of participants in favor of accepting refugees. The topic of health did not arise in other methods of analysis of our material. Nevertheless, it might have been related to the public debate about refugees in Poland, although especially its prejudiced iterations were delivered by certain politicians, who claimed that refugees pose danger as carriers of diseases (Gera, 2015).

## Triangulation, integration, and methods comparison

In the current research, we used various methods to explore measuring attitudes towards outgroups on the example of attitudes towards refugees. The results of the *Attitudes Towards Refugees Scale* showed that the participants’ attitudes expressed via closed-ended statements were generally negative and participants were opposed to hosting refugees in Poland. The results of the manual content analysis of the answers to the open-ended question revealed a more positive view: roughly one third (32% and 38%) of the participants opposed accepting refugees. Although the results from the scale were strongly correlated with the results coded as the *Accept refugees* category, the qualitative analysis of the answers allowed us to observe many conditions under which the participants were willing to accept refugees. Such conditions were: an expectation that the refugees will assimilate, that they should work, or that they should be controlled by the state. Whereas the closed-ended answers and the percentages of the coded open-ended answers only showed that the attitudes were more negative in Study 2 than in Study 1, the content analysis of the open-ended questions also showed how the discourse and the topics mentioned changed between Study 1 and 2. For example, the main topic that the refugees should work present in Study 1 was less prominent in Study 2. Instead, the participants concentrated on the fact that Poland should accept only a certain number of refugees and a certain kind of people. This is in line with the extensive research on agenda setting, which shows that people emphasize in their responses what is on the media and this can shift even in a much shorter time than a year (e.g., Feezell, 2018).

The subsequent analysis conducted using MEH—an automated tool to extract themes from the text—yielded fewer themes than the manual coding, but the themes to some extent reflected some of the themes from the manual analysis. However, they were structured differently, as they often mixed positive statements (e.g., *give them a chance to work*) and negative statements (e.g., *put them in work camps*) as long as they were about the same topic (here: work). Manual coders observed that these were on the same topic, but intuitively divided them according to the supportive or oppressive intentions that they saw behind each statement. The results of the automated sentiment analysis with Sentiment Analyzer and LIWC provided us with a comparison of emotion words used in Studies 1 and 2 and by participants for and against accepting refugees. LIWC and Sentiment Analyzer to some extent showed that the general valence, or amount of positive emotions, was higher in Study 2 than in Study 1, which was contrary to the answers on the acceptance scale or to the percentages from the coded open-ended answers. The results from the Sentiment Analyzer combined with those from the manual coding were more detailed and showed rather that the response texts were more polarized and intense (more positive and less neutral) in Study 2 than in Study 1. From the thematic analysis and the manual coding, we saw that participants in Study 2 talked more about helping refugees in their countries or about accepting only a certain kind of people than in Study 1. On a linguistic level, words related to helping and accepting are positive, but how they were used was actually expressing more negative attitudes, for example, many participants said that Poland should send humanitarian help outside instead of accepting refugees to the country. In line with the other results, Sentiment Analyzer results also showed more sadness and harm in Study 2 than in Study 1. Using LIWC allowed us to compare more than just valence and emotions, and the results also showed that participants in Study 1 mentioned work more often than participants in Study 2, which reflects the manual coding and MEH analysis. In general, the results of LIWC and Sentiment Analyzer show advantages of the relatively quick and easy-to-use dictionary tools, but also the limitations of using and interpreting data based on one type of analysis only.

## General discussion

The goal of the current research was twofold. First, we wanted to compare and integrate different methods of assessing attitudes towards outgroups, particularly to refugees. Second, we wanted to compare various methods of analyzing open-ended answers: manual content analysis and three automated text analysis tools (MEH, Sentiment Analyzer, and LIWC).

The results of the different methods partly converged, but each method also afforded a view of the data from a different angle. This conclusion is not historically new (see e.g., Geer, 1991; Krosnick, 1999). Furthermore, also other researchers have called for using open-ended questions, as these allow us to learn from participants’ ideas that researchers themselves would not come up with (e.g., Geer, 1991; Haddock & Zanna, 1998). In the current research we extend this with an observation that with open-ended questions one learns about explanations of people’s views and attitudes. These explanations are crucial to understanding attitudes, as basing an interpretation solely on closed-ended answers could lead researchers to interpret these attitudes incorrectly. With our research, we remind of these important, but in the last years largely forgotten, statements. We also show how to combine methods with the help of modern tools that allow for a relatively fast analysis of a large body of open-ended answers. We tested various tools on the same material and the researchers can choose which of the methods they want to use in their studies. If one decides to use more than one method or even all of them at the same time, it is important to thoughtfully integrate and interpret them. When the methods produce convergent results, the task of integrating them is relatively easy. But what if the methods generate ambiguous or even contradictory results? In the following section, we discuss our findings showing how the results coming from different methods can be integrated, how they complement each other, and what to do when the results differ across methods.

### Comparing and integrating results of different methods

In the current research, closed-ended answers of the same participants were more negative than their open-ended answers. We think that this difference can be attributed to the format of the questions and to the fact that attitudes towards refugees are ambivalent, complex, and not well defined. When asked in an open format, participants can better explain their views and follow less the social norm (Connor Desai & Reimers, 2019; Frew et al., 2003). When integrating such results one must take into account the qualitative content of the open-ended answers. In our case, participants forced to answer on a scale chose to be more conservative in their answers, but when they could show the complexity of the issue and of their views, they stated more conditional answers as to not only *whether* to accept refugees, but *how* it should be done.

For the open-ended answers, we analyzed exactly the same content, so the differences we encountered in the results stem from the specific analysis methods and tools that we used. The manual analysis allowed for different levels of coding and for detecting indirect statements, irony, or negation. The results of MEH also produced some of the themes that emerged in the manual coding. These results agreed with each other to some extent and MEH could be seen as an alternative and quicker method of extracting meaning and creating themes. However, some of the themes were different as automatic meaning extraction does not take into account the valence of the answers. This was visible, for example, in the MEH-generated theme about learning the language, where some participants were writing about offering help, including language courses, others were stating that refugees should be forced to learn Polish, and still others were skeptical whether refugees would be able or willing to learn Polish. In order to integrate these partly disparate results, it is crucial to understand the content of the themes generated with a MEH analysis. To do so, it is important to look not only at the words in each theme but to carefully read the highly-loading responses from each extracted category (see also Ikizer et al., 2019). Researchers studying attitudes or other strongly valanced phenomena should either use MEH very carefully or use it in parallel with manual coding of at least some portion of the data.

Further cues to participants’ attitudes towards refugees came from the exact words they used in their written responses. Most importantly, automated text analyses allowed us to identify the emotional tone of the answers. They also provided an overview of the psychological constructs that surfaced while participants were expressing their views. The results of Sentiment Analyzer- and LIWC-based analyses indicated that on the linguistic level, participants emotions were more extreme and also more positive about refugees in Study 2 than in Study 1. Interestingly, the more positivity in Study 2 findings from two different software programs were not in line with the results from the closed-ended answers or from the manual coding, with these last two revealing more negative attitudes towards refugees in Study 2 compared to Study 1. How to reconcile these results? When the results were combined with the information from manual coding, the findings showed that it was mainly that in Study 2 participants expressed their views more intensely, more emotionally in general. Congruent with the above and with the results of manual coding, Sentiment Analyzer results showed more sadness and harm in Study 2 than in Study 1. Other Sentiment Analyzer and LIWC results concerned specific themes. These largely corresponded to what we found in manual coding as well as in automatic meaning extraction with MEH. In particular, LIWC, as a more comprehensive tool than Sentiment Analyzer, evidenced performance that was overall consistent with that of human coders. Furthermore, Sentiment Analyzer results showed that the responses of the participants whose answers were manually coded as accepting of refugees were also more positive, as shown by the automated analyses, than answers of participants who were against refugees. Overall, LIWC and Sentiment Analyzer are easy-to-use and time-efficient tools that complement the results from closed-ended questions. We, however, recommend using such fully automated tools in parallel with methods that capture the meaning and context of the responses.

To deepen our understanding of the participants’ attitudes and to compare the methods, we correlated the results from the closed-ended answers with the variables from the coding of the open-ended answers (as recommended in Onwuegbuzie & Teddlie, 2003). We also compared the means on the closed-ended scale for participants who mentioned or did not mention each given topic in their open-ended answer. The results of the correlations and mean comparisons showed similar results and can be treated as alternative methods of showing how the results of coded open-ended answers relate to closed-ended answers. Some correlations and mean comparisons merely showed a convergence of these methods with manual coding (e.g., participants who had positive attitudes towards refugees were also more supportive in their spontaneous answers), but some were surprising given the previous work on attitudes towards refugees conducted using closed-ended questions. However, these results were understandable and rational given the results of our open-ended questions. Similar results were obtained when combining codes from manual analysis with Sentiment Analyzer and LIWC variables. Overall, combining methods and letting participants express their opinion in their own words gives researchers insights into the reasoning behind the given answers and allows for a better understanding of attitudes.

### Advantages and disadvantages of closed- and open-ended questions

In the current article, we showed the advantages of using open-ended questions for measuring attitudes and encouraged researchers to combine open- and closed-ended questions in their research. However, one should also consider the weaknesses and limitations of open-ended questions. While open-ended questions provide richer, more nuanced responses, it is much more difficult to get people to respond to them than to respond to closed-ended questions. Additionally, sometimes open-ended responses may just not be necessary. If one is measuring attitudes that are well-formed and that participants are certain about, it might not be necessary to use open-ended questions. Similarly, if one is conducting a series of studies and sees that over time the content of the answers stays similar, in the later studies it might not be needed to bother participants with responding to open-ended questions.

In our research, we compared different automated text analysis methods. They all are quicker than manual coding, but they also require some time investment. We devoted some time to pre- or post-processing (MEH: checking the themes; Sentiment Analyzer: correction of spelling before the analysis; LIWC: translation from English into Polish). However, some of these corrections, such as correcting spelling, are not obligatory; the quantity can make up for quality. Researchers who analyze many thousands of, for instance, tweets do not correct anything or they use only standard corrections of the most popular mistakes (Ikizer et al., 2019). This means losing some data, but with a very large dataset this does not constitute a big problem.

When it comes to the MEH analyses, they were very useful, objective, and relatively time-efficient. However, some features of the method itself influenced the results. Most importantly, such automated analyses as MEH detect the occurrence and co-occurrence of words without taking into account negation or context. Consequently, texts within a given theme may mention the same words and concepts, but can be expressing opposite intentions. Furthermore, the longer the text, the better MEH can classify it, so as a rule, the texts that are the highest-loading on a specific theme are rather the longer ones. Human coders are able to reliably extract themes and their valence manually even from short texts. All that said, we expect that the next years might bring new tools for sentiment analysis (e.g., similar to VADER, Hutto & Gilbert, 2014) that will overcome some of the limitations of the current tools.

## Conclusions and implications

In the current research, the use of various methods applied to the same material allowed for contrasting them and looking at the advantages and limitations of each one. The manual coding allowed for the most detailed and context-sensitive analysis. This was manageable with the current dataset, but when working with large amounts of data collected automatically (e.g., from Twitter) manual coding would be impractical. The automated text analyses provided some approximation of the manual coding. However, we recommend using more than one of such tools at the same time. The results of each method separately converged only to some extent with each other and with the manual coding. Using two (or more) such tools would help diminish problems inherent to the automated methods, such as being either valence- or context-insensitive, or analyzing valence but focusing less on the topics mentioned. We can recommend using automated tools for large datasets, but with an additional manual analysis of parts of the most representative answers.

A direct real-world implication of our results is that instead of a simple yes or no to accepting refugees, there should be more space for discussion as to who should be accepted and how could the newcomers be integrated into the society. In order to do this, researchers and policymakers could use a broad array of methods of assessing and analyzing attitudes towards outgroups.

## Data Availability

The data and materials for all studies as well as the codebooks used for manual coding of answers are available at https://osf.io/3naj5/?view_only=f849eee116a5447db19290160f00ba39.

## Footnotes

1 The research company that collected the sample uses only two gender/sex categories: “man” and “woman.” In Polish there is just one word for both gender and sex: “płeć.”

2 In general, online panels suffer from coverage bias (i.e., do not include the offline population) and self-selection bias (i.e., include only people who sign up themselves for the panels). Our findings are thus generalizable to the Polish population that uses the Internet.

3 In Study 1, before the open-ended question, the participants were shown four scenarios describing a refugee and a few questions related to the scenarios. The scenarios had no effect on either the closed- or the open-ended responses, so we combined the data across the four conditions. In Study 2, we included a measure of dehumanization to deepen the understanding of the topic of forced work that emerged in Study 1. However, because in Study 2 this topic was not as prevalent as in Study 1, we excluded it from the analyses reported here. We provide all the data and results of the above analyses under the following link: https://osf.io/3naj5/?view_only=f849eee116a5447db19290160f00ba39.

4 Sentiment Analyzer was not accessible through CLARIN’s web at the time of conducting our studies; instead, the analyses were carried out through personal communication with the tool’s creators (A. Janz, personal communication, February 7 & March 20, 2018).

5 Closed-ended answers in polls and in our closed-ended scale did not have the *other* category, so when counting only the *yes/no/don’t know* responses, the percentage of the *yes* answers would be even higher.

6 One should be cautious in interpreting the results of the *t*-tests due to the unequal distributions of the “code present” vs. “code absent” groups. These analyses are, however, replicated on both studies and are per se exploratory in nature.

## Code availability

Code used to analyze the data is available at https://osf.io/3naj5/?view_only=f849eee116a5447db19290160f00ba39.
